# Label-Free Detection and Spectrometrically Quantitative Analysis of the Cancer Biomarker CA125 Based on Lyotropic Chromonic Liquid Crystal

**DOI:** 10.3390/bios11080271

**Published:** 2021-08-11

**Authors:** Hassanein Shaban, Mon-Juan Lee, Wei Lee

**Affiliations:** 1 Institute of Imaging and Biomedical Photonics, College of Photonics, National Yang Ming Chiao Tung University, Guiren District, Tainan 71150, Taiwan; hassanein.shaban@sci.asu.edu.eg; 2 Department of Basic Science, Faculty of Engineering, The British University in Egypt, El Sherouk City 11837, Egypt; 3 Department of Bioscience Technology, Chang Jung Christian University, Guiren District, Tainan 71101, Taiwan; 4 Department of Medical Science Industries, Chang Jung Christian University, Guiren District, Tainan 71101, Taiwan

**Keywords:** lyotropic chromonic liquid crystal, label-free biosensor, optical biosensor, protein assay, immunoassay, transmission spectrometry

## Abstract

Compared with thermotropic liquid crystals (LCs), the biosensing potential of lyotropic chromonic liquid crystals (LCLCs), which are more biocompatible because of their hydrophilic nature, has scarcely been investigated. In this study, the nematic phase, a mesophase shared by both thermotropic LCs and LCLCs, of disodium cromoglycate (DSCG) was employed as the sensing mesogen in the LCLC-based biosensor. The biosensing platform was constructed so that the LCLC was homogeneously aligned by the planar anchoring strength of polyimide, but was disrupted in the presence of proteins such as bovine serum albumin (BSA) or the cancer biomarker CA125 captured by the anti-CA125 antibody, with the level of disturbance (and the optical signal thus produced) predominated by the amount of the analyte. The concentration- and wavelength-dependent optical response was analyzed by transmission spectrometry in the visible light spectrum with parallel or crossed polarizers. The concentration of CA125 can be quantified with spectrometrically derived parameters in a linear calibration curve. The limit of detection for both BSA and CA125 of the LCLC-based biosensor was superior or comparable to that of thermotropic LC-based biosensing techniques. Our results provide, to the best of our knowledge, the first evidence that LCLCs can be applied in spectrometrically quantitative biosensing.

## 1. Introduction

**Figure 1:**
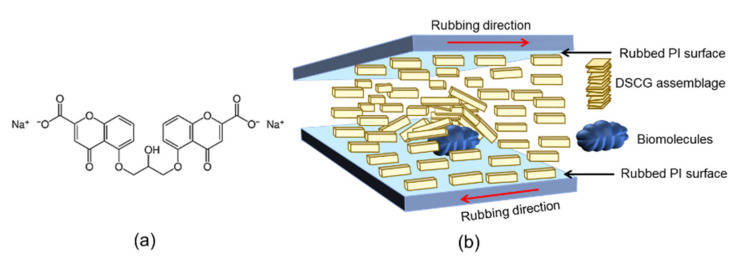
LCLC-based biosensing with planarly aligned DSCG LCLC. (**a**) The chemical structure of DSCG and (**b**) an illustration simulating the disturbance in the planarly aligned columnar stacks of DSCG in the presence of biomolecules.

Most liquid crystal (LC)-based biosensing techniques reported to date employ thermotropic LCs, especially the rod-like nematic 4-cyano-4′-pentylbiphenyl (5CB), as the predominant sensing medium [1,2,3,4]. Building on technologies of 5CB biodetection at the LC–water interface, in the form of LC film on water, LC droplets, and LC emulsions [5,6], or the LC–glass interface with an LC cell configuration [7], various biosensors utilizing other types and phases of LCs, such as cholesteric LCs [8], dye LCs [9], and dual-frequency LCs [10], were developed to transduce the optical, electro-optical, and dielectric signals produced by biomolecules or biomolecular interactions [11]. Nevertheless, the biosensing application of lyotropic LCs is relatively scarce due possibly to the concentration-dependent polymorphic phase transitions and the lack of effective signal transduction approaches. Lyotropic chromonic liquid crystals (LCLCs) are a unique class of lyotropic LCs most frequently investigated in biosensing. LCLCs consist of water-soluble compounds characterized by a plank-like structure with a polyaromatic core linked to plural hydrophilic side groups (Figure 1a) [12]. When the drug disodium cromoglycate (DSCG) is dissolved in water, the nematic LCLC phase can be observed at room temperature (23 °C) at concentrations ranging from 12 to 17 wt%, with the viscosity strongly dependent on the DSCG content [13]. The DSCG molecules form rod-like columnar assemblages through π–π stacking of the hydrophobic polyaromatic structure, which are separated by a distance of ∼3.4 Å by virtue of the ionic repulsion between hydrophilic side groups [14]. Depending on the concentration and temperature, the self-assembled columnar stack of DSCG exhibits a lateral separation of ∼35 Å to 42 Å and lengthens with increasing DSCG concentration, contributing to a higher birefringence and lower viscosity than other lyotropic liquid crystals [14,15,16].

Owing to its low cytotoxicity and hydrophilic nature, the self-organization and anisotropic properties of the nematic phase of DSCG were utilized in the dynamic detection of motile bacteria [17,18,19,20]. DSCG is more biocompatible with viruses and mammalian cells, allowing the vesicular stomatitis virus to remain active and replicate in human cervical epithelial carcinoma cells, in contrast to the zwitterionic surfactant C14AO, an amphiphilic lyotropic liquid crystal that results in virus inactivation and cell death [21]. In biomolecular detection, DSCG is considered congruent with molecular interactions without affecting the binding activity of anti-immunoglobulin (IgG) antibodies to immobilized human IgG [15]. The intensity of transmitted light observed under a polarizing optical microscope (POM) was enhanced when the ordered planar alignment of nematic DSCG was disrupted by the aggregation of streptavidin-coated latex beads in the presence of anti-streptavidin antibodies, but not in the absence of immunocomplex formation [22]. This implies that biodetection with the nematic phase of LCLCs is governed by a similar principle to thermotropic LCs, in which the level of disturbance caused by the analyte transduces to the amplitude of the resulting optical signal (Figure 1b).

The cancer antigen 125 (CA125) is expressed as a membrane glycoprotein on the cell surface of ovarian, breast or gastric cancer cells, but may be released in soluble form into the blood. The reference level of CA125 in a blood sample is 30–35 U/mL, beyond which indicates a higher risk of cancer progression [23]. Conventional methods for the detection of CA125 are sandwich assays, a type of immunoassay in which CA125 reacts specifically with a capture antibody immobilized on a solid substrate and a detection antibody with fluorometric or colorimetric labeling. The procedure of such label-based detection is time-consuming and the nonspecific binding of the labeled antibody may lead to false-positive signals. As part of an effort to develop rapid screening and cost-effective point-of-care diagnostics, novel techniques for the label-free detection of CA125 have been actively investigated, including electrochemical immunosensors [24,25], aptasensors based on field-effect transistors [26], and impedimetric immunosensors with gold nanostructured screen-printed electrodes [27]. In biosensing on the basis of thermotropic LCs, our previous work demonstrated the feasibility of a nematic LC of high birefringence as well as an LC–photopolymer composite in highly sensitive label-free CA125 immunodetection [28,29,30]. Because current LC-based biosensing technologies rely heavily on the optical response derived from the LC texture observed under a POM, which can only provide qualitative or semiquantitative results, we incorporated transmission spectrometric analysis in a label-free CA125 immunoassay employing dye-doped LC as the sensing medium to enhance detection sensitivity and to elaborate a more accurate quantitative strategy [31].

In this study, an LCLC-based quantitative protein biosensor and an immunosensor utilizing the nematic phase of DSCG as the sensing medium were developed in conjunction with transmission spectrometry. The sensing platform was fabricated by sandwiching the aqueous solution of DSCG between a pair of glass substrates coated with polyimide (PI) to promote planar alignment. The optical signal derived from the biological analyte—either the common protein standard BSA or the tumor marker CA125 captured by the anti-CA125 antibody—immobilized at the LC-glass interface was analyzed by measuring the transmission spectra of the sandwich cell placed between parallel or crossed polarizers. The spectrometric analysis thus established provides not only absolute quantitation of analyte concentration from the wavelength-dependent optical response, but also the basis for the optimization of detection sensitivity and limit of detection.

## 2. Materials and Methods

### 2.1. Materials

Optical-grade glass substrates (22 × 18 × 1.1 mm) were purchased from Ruilong Glass, Miaoli, Taiwan. The commercial PI SE-150 (0821) from Nissan Chemical was utilized to prepare a planar alignment layer on a glass substrate. Both DSCG and BSA were provided by Sigma-Aldrich. At 14 wt% in DI water, DSCG exhibits the nematic phase, and the phase transition temperature from the nematic to isotropic phase was determined to be ca. 27.5 °C. Recombinant human CA125/MUC16 protein and anti-CA125 antibody employed in the LCLC-based immunoassay were manufactured by R&D Systems and Santa Cruz Biotechnology, respectively.

### 2.2. Preparation of PI-Coated Glass Substrates

**Figure 2:**
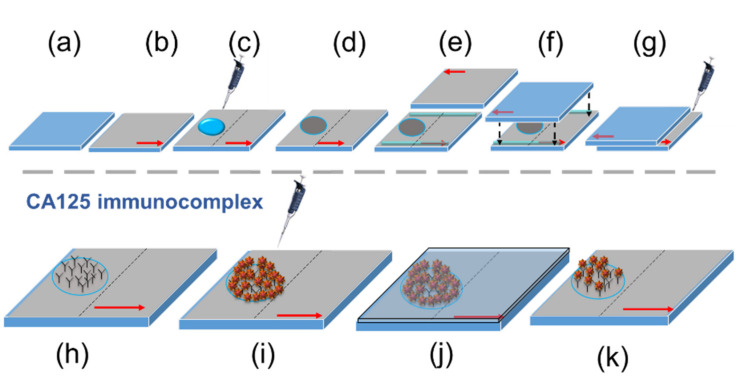
The LCLC-based protein detection and immunoassay platform. A cleaned optical glass substrate (**a**) was coated with PI SE-150 and rubbed unidirectionally (**b**). A 30 μL aqueous solution of BSA or anti-CA125 antibody was dispensed on the rubbed PI-coated surface (**c**) and allowed to dry at 30 °C for 30 min (**d**). After rinsing with DI water, the 15-μm spacer was mixed with the epoxy resin AB glue and distributed on two parallel edges of the optical glass substrate (**e**), followed by LC cell assembly with another protein-free PI-coated glass substrate with antiparallel rubbing direction (**f**). Finally, the LC cell was filled with 14-wt% DSCG solution for further optical measurements (**g**). For the CA125 immunoassay, immobilized anti-CA125 antibodies (**h**) were reacted with CA125 by dispensing 30 μL of the CA125 protein solution on the glass substrate (**i**), which was covered with a cover glass to allow immunoreaction to occur for 30 min (**j**). After removing the cover glass, the glass substrate was rinsed with DI water (**k**), followed by LC cell assembly as described in (**e**–**g**).

Cleaned glass substrates were spin-coated with PI SE-150 (0821) at 2000 rpm for 30 s, followed by 6000 rpm for 60 s. The PI-coated substrates were then soft-baked at 85 °C for 10 min and successively hard-baked at 240 °C for 60 min. Unidirectional rubbing was performed 30 times on the PI-coated surface to enhance the planner anchoring strength, as shown in Figure 2a,b.

### 2.3. Construction of the LCLC-Based Protein Detection Platform

The surface of the rubbed PI-coated substrates was dissected into two areas: one for immobilizing biomolecules and detecting the analyte, and the other for monitoring background signals in the absence of immobilized proteins. BSA or anti-CA125 antibody was dispensed at 30 µL/spot with a Gilson Pipetman G P200G micropipette on one of the PI-coated glass substrates, which was dried at 30 °C for 30 min and rinsed with DI water to remove non-adsorbed biomolecules (Figure 2c,d). The circular area of the immobilized protein thus formed covered the entire cross section of the propagating light beam during the optical measurement. To assemble the LC cell, rod spacers of 15 μm in diameter were mixed with a small amount of the epoxy resin AB glue and distributed on two parallel sides of the glass substrate with immobilized biomolecules, which was covered with another protein-free PI-coated substrate with antiparallel rubbing direction (Figure 2e,f). The assembled LC cell was allowed to dry for 5 min at room temperature before further experiments were carried out. The cell gap of the empty LC cell was determined by optical interferometry with the Ocean Optics HR2000+ high-resolution USB fiber-optic spectrometer [32]. Afterwards, the LC cell was filled with 10-μL aqueous solution of 14-wt% DSCG by suction and sealed with AB glue (Figure 2g).

### 2.4. LCLC-Based ca125 Immunoassay

For the CA125 immunoassay, the immobilized anti-CA125 antibody was further reacted with the CA125 protein by dispensing 30 µL of the CA125 solution on the glass substrate, followed by covering the glass substrate with a cover glass so that the entire glass surface was in contact with the CA125 protein solution (Figure 2h–j). After reacting for 30 min, the cover glass was removed, and the glass substrate was rinsed with DI water to eliminate unbound CA125 (Figure 2k). LC cell assembly was then performed as described in Section 2.3 as well as illustrated in Figure 2e–g.

### 2.5. Transmission Spectrometric Measurements

**Figure 3:**
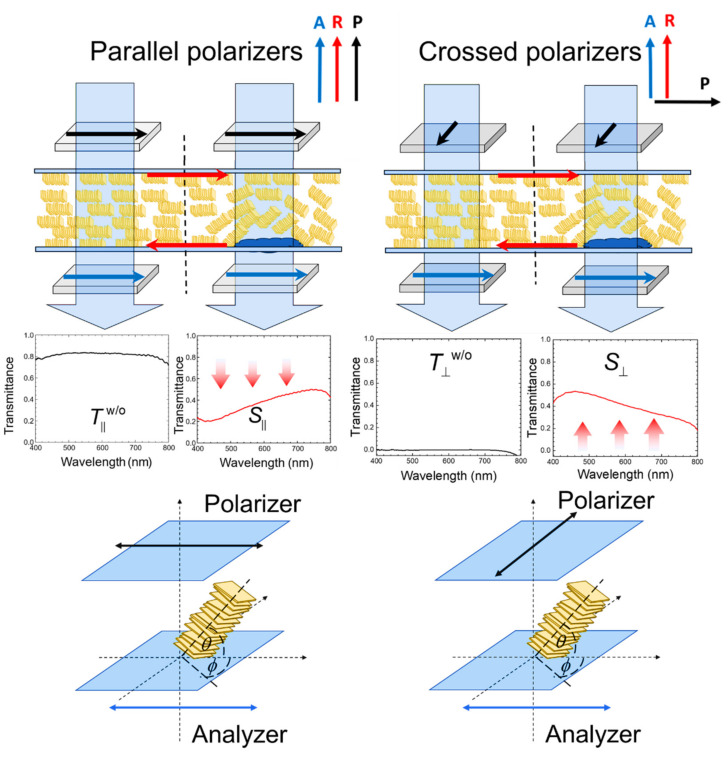
Transmission measurements with parallel and crossed polarizers in the LCLC-based biosensor. When biomolecules are immobilized at the LC–glass interface, leading to disturbance in the planar alignment of LCLC, transmittance decreases when analyzed with parallel polarizers (left panel) and increases when analyzed with crossed polarizers (right panel).

Transmission spectra in the wavelength range of 400–800 nm were acquired with an Ocean Optics HR2000+ high-resolution fiber-optic spectrometer equipped with an Ocean Optics HL-2000 tungsten halogen as the light source. During spectral measurements, the LC cell was placed between two linear polarizers, with its rubbing direction parallel to the transmission axis of the lower polarizer or the analyzer, as shown in Figure 3. The transmission axis of the upper polarizer was oriented by either 0° or 90° measured from that of the lower polarizer, forming two modes of spectrometric detection with parallel or crossed polarizers, respectively.

### 2.6. Optical Texture Observation

BSA immobilization and formation of the CA125 immunocomplex were enabled as described in Section 2.3 and Section 2.4 except that BSA or anti-CA125 antibody was immobilized at 3 μL/spot to form a 3 × 3 array on the PI-coated glass substrate. The optical texture of the DSCG aqueous solution was observed under a POM (Olympus BX51, Tokyo, Japan) with the rubbing direction of the LC cell parallel to one of the transmission axes of the crossed polarizers.

## 3. Results and Discussion

### 3.1. Principle of Detection by Transmission Spectrometry in the LCLC-Based Biosensor

When transmission spectrometric analysis is performed with parallel polarizers, the transmittance or normalized intensity of the transmitted light, *I*_‖_, can be formulated as:

(1) $$I_{∥}=1-\sin^{2}(2\phi )\cdot \sin^{2}(\delta /2)$$

where *ϕ* is the azimuthal angle of the average molecular axis of LC, or the LC director, with respect to the transmission axis of the analyzer, and *δ* is the phase retardation [13]. The phase retardation can be, in turn, calculated as δ=2πdΔn/λ, where *d* is the cell gap, *λ* is the wavelength of the incident light, and Δ*n* is the LC birefringence. By definition, $\Delta n\equiv n_{e\mathrm{ff}}-n_{⊥}$, where *n*_⊥_ is the refractive index of the LC when the electric field of the incident light is perpendicular to the LC director, while *n*_eff_ is the effective refractive index given by:

(2) $$n_{\mathrm{eff}}=\frac{n_{⊥}n_{∥}}{\sqrt{n_{∥}^{2}\sin^{2}\theta +n_{⊥}^{2}\cos^{2}\theta }},\;$$

in which *n*_‖_ is the refractive index of the LC when the vibration direction of the electric field component of the impinging light is parallel to the LC director, and *θ* is the pretilt angle between the director and the substrate plane. In the absence of an analyte, the LC director is parallel to the transmission axes of the polarizers (*ϕ* = 0°) in the parallel polarizer scheme so that *I*_‖_ is at its maximum (*I*_‖_ = 1). When the orientation of LCs is disturbed by biomolecules immobilized at the LC–glass interface, *ϕ* grows (*ϕ* > 0°), resulting in a decrease in *I*_‖_. As *ϕ* = 45°, Equation (1) becomes *I*_‖_ = cos^2^ (*δ*/2), and no optical transmission is observed at dΔn/λ=m+1/2 (where *m* = 0 or a positive integer).

Conversely, when spectral measurements are performed with crossed polarizers in the absence of an analyte, the transmittance or normalized intensity of the transmitted light, *I*_⊥_, can be expressed as:

(3) $$I_{⊥}=\sin^{2}(2\phi )\cdot \sin^{2}(\delta /2),$$

which is therefore at its minimum when *ϕ* = 0°. In the presence of an analyte when *ϕ* > 0°, *I*_⊥_ increases and maximum optical transmission results with *ϕ* = 45° such that Equation (3) becomes $I_{⊥}=\sin^{2}(\delta /2)$ at dΔn/λ=m+1/2 (where *m* = 0 or a positive integer). Accordingly, when biomolecules are present on the lower PI-coated glass substrate of the LC cell, the planar alignment of LC molecules in contact with and in close proximity to the analyte is disturbed, and the wavelength-dependent optical signal obtained with either parallel or crossed polarizers is generated at non-zero azimuthal angles (*ϕ* ≠ 0) and reaches its maximum at *ϕ* = 45° (Figure 1 and Figure 3). Moving toward the upper PI-coated substrate of the LC cell, where no biomolecules are immobilized, the homogeneous alignment of LC molecules is preserved. The LC molecules in the region with immobilized biomolecules are therefore in the twisted configuration, surrounded by those in perfectly planar state in areas without the analyte.

Moreover, the value of *θ* varies between *θ* = 0°, corresponding to the planar alignment of the LC at maximum birefringence, and *θ* = 90°, signifying the vertical alignment of the LC with vanished birefringence (Δ*n* = 0). At *θ* = 0°, the deviation of *ϕ* from zero (*ϕ* > 0) strongly affects *I*_‖_ and *I*_⊥_, which, in contrast, remain unaltered by *ϕ* at *θ* = 90°. According to the molecular theory of surface tension, the surface tension of the solid substrate (*γ*_c_) is much greater than the surface tension of the planarly aligned LC (*γ*_l_). Therefore, in the presence of an analyte when *ϕ* > 0, the LCLC may be directed by surface tension to become reoriented outside the unidirectional “grooves” produced by rubbing. In addition, the twist elastic constant *K*_22_ of LCLC is much smaller than the splay and bend elastic constants, *K*_11_ and *K*_33_, respectively, supporting that the orientation of LCLC molecules tends to deviate azimuthally in the substrate plane in response to external stimuli [33].

To quantitate the optical signal in correlation with the amount of analyte and to eliminate false-positive or nonspecific background noise, the reduced transmittance parameters obtained with parallel and crossed polarizers, *T*_‖_ and *T*_⊥_, are defined as:

(4) $$T_{\mathrm{ll}}=\frac{S_{\mathrm{ll}}-T_{⊥}^{w/o}}{T_{\mathrm{ll}}^{w/o}-T_{⊥}^{w/o}}$$

and

(5) $$T_{⊥}=\frac{S_{⊥}-T_{⊥}^{w/o}}{T_{\mathrm{ll}}^{w/o}-T_{⊥}^{w/o}}$$

respectively, where $S_{\mathrm{ll}}^{}$ and *S*_⊥_ stand for the respective transmittance in the presence of analyte molecules measured with parallel and crossed polarizers, and $T_{\mathrm{ll}}^{w/o}$ and $T_{⊥}^{w/o}$ are the transmittance in the absence of an analyte measured with parallel and crossed polarizers, respectively.

### 3.2. LCLC-Based Spectrometric Quantitation of BSA

**Figure 4:**
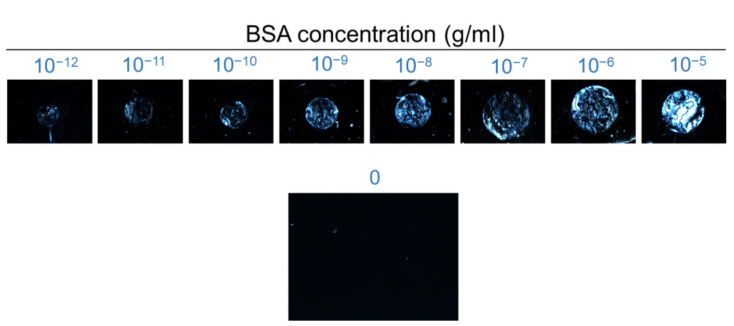
Optical textures of planarly aligned LCLC in the presence of BSA. The LCLC-based protein detection was performed with an aqueous solution of 14-wt% DSCG as the sensing medium in contact with 10^−12^–10^−5^ g/mL BSA immobilized on the PI-coated glass surface. The micrograph at the bottom displays a completely dark texture of a reference of dried DI water containing no BSA.

In this work, the protein detection capability of the LCLC-based biosensor was demonstrated with a globular protein, the common protein standard BSA, and an antibody, the anti-CA125 antibody against the cancer biomarker CA125. Various concentrations of BSA were first immobilized on the PI-coated glass substrate, followed by LC cell assembly and optical texture observation under a POM with crossed polarizers. In the absence of BSA, DSCG was planarly aligned by the planar alignment agent PI, and the optical texture was completely dark, as depicted in Figure 4. As BSA accumulated at the LC-glass interface, light leakage increased with increasing concentrations of BSA (Figure 4). A similar dark-to-bright transition was also reported in the detection of BSA by homeotropically aligned DSCG [34]. However, DSCG tends to reorient from the homeotropic to the planar state over time [35]. Indeed, because of the low anchoring energy of conventional alignment agents and rubbing methods, it is more difficult to align hydrophilic LCLCs than hydrophobic thermotropic LCs, especially for the homeotropic alignment of LCLCs [36,37]. Our method of planar alignment with rubbed PI expectedly offered stable alignment with promoted anchoring strength and uniform surface [38].

**Figure 5:**
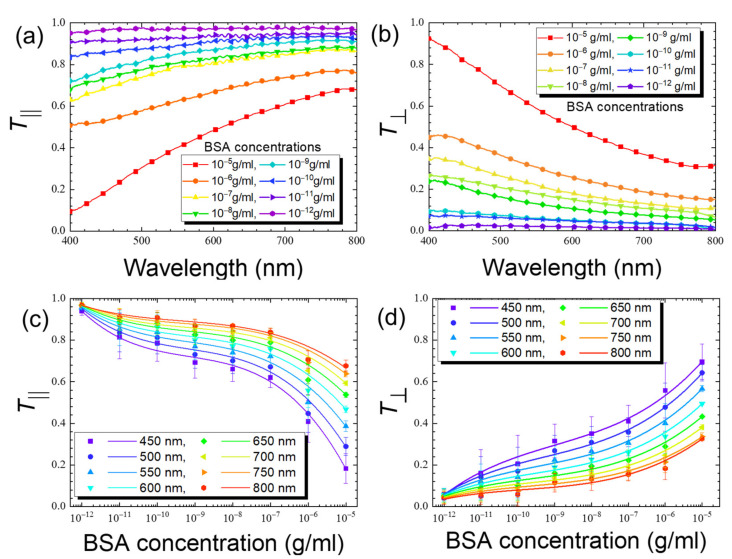
Reduced transmission data of LCLC with 14-wt% DSCG in the presence of BSA at various concentrations ranging from 10^−12^ to 10^−5^ g/mL. The reduced transmittance parameters (**a**) *T*_‖_ and (**b**) *T*_⊥_ in the wavelength range of 400–800 nm at various BSA concentrations and (**c**) *T*_‖_ and (**d**) *T*_⊥_ plotted against BSA concentration at selected wavelengths of 450, 500, 550, 600, 650, 700, 750, and 800 nm for quantitative purpose. *T*_‖_ and *T*_⊥_ correspond to the respective parallel polarizer and crossed polarizer schemes for the spectrometric detection, respectively. Error bars represent standard deviations (*n* ≥ 3).

Transmission spectrometric measurement with parallel and crossed polarizers was performed to explore methods for the absolute quantitation of the optical signal (Figure 5). As shown in Figure 5a, when parallel polarizers were utilized, reduced transmittance in the wavelength range of 400–800 nm decreased with increasing concentrations of BSA. At 10^−12^ g/mL, *T*_‖_ was close to its maximum value of unity, which corresponds to the planar state in the absence of an analyte, suggesting that the amount of BSA was insufficient to significantly alter the orientation of DSCG assemblages in water. As the concentration of BSA increased, *T*_‖_ decreased in a concentration-dependent manner, indicating an increase in the azimuthal angle and the deviation of the DSCG alignment from the rubbing direction. On the other hand, when crossed polarizers were applied, *T*_⊥_ at 10^−12^ g/mL BSA approached its minimum of 0, which represents unperturbed planar alignment, and the increase in the amount of BSA led to elevated *T*_⊥_ in a concentration-dependent manner (Figure 5b).

When the reduced transmittance *T*_‖_ (*T*_⊥_) at selected wavelengths was plotted against BSA concentration, a negative (positive) correlation was found (Figure 5c,d). While linear correlation can be ascertained in a narrower range of concentrations, the calibration curve for the entire BSA concentration range (10^−12^–10^−5^ g/mL) was obtained through the third-order polynomial curve fitting that describes *T*_‖_ or *T*_⊥_ as a function of the logarithm of BSA concentration. The quality of the curve fitting, as evaluated by calculating the coefficient of determination, *R*^2^, is satisfactory at ~0.99 at all wavelengths examined. One can see from (Figure 5c,d) that a more significant decrease in *T*_‖_ and increase in *T*_⊥_ with increasing BSA concentrations, respectively, were observed for a shorter wavelength, especially at 450 nm. These results indicate that the sensitivity of the LCLC-based spectrometric protein quantitation can be fine-tuned through the wavelength of the incident light, and the highest sensitivity was found at the shortest wavelength of 450 nm examined in this study. This can be explained by the effective phase retardation *δ*, which was larger at a shorter wavelength, in accordance with the wavelength-dependent birefringence of DSCG [13]. The limit of detection (LOD) was calculated using the following equation:

(6) $$\mathrm{LOD}=\frac{3s}{m}$$

where *s* is the standard deviation of the *y*-intercept and *m* is the slope of the calibration curve obtained by linear regression [39]. Consequently, the LOD obtained with parallel polarizers (LOD_‖_) and that with crossed polarizers (LOD_⊥_) at all measured wavelengths ranged between 10^−11^ and 10^−10^ g/mL BSA, with LOD_‖_ and LOD_⊥_ values at 450 nm calculated as 2.4 × 10^−10^ and 6.0 × 10^−11^ g/mL BSA, respectively.

### 3.3. LCLC-Based Spectrometric Quantitation of the Anti-CA125 Antibody

**Figure 6:**
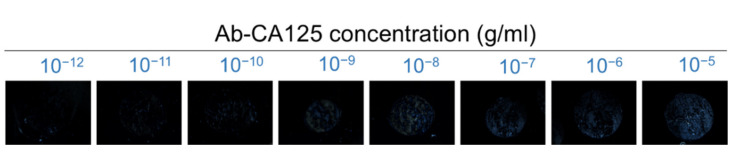
Optical textures of planarly aligned LCLC in the presence of the anti-CA125 antibody. The LCLC-based protein detection was performed with an aqueous solution of 14-wt% DSCG as the sensing medium in contact with 10^−12^–10^−5^ g/mL anti-CA125 antibody immobilized on the PI-coated glass surface.

The effect of the concentration of the anti-CA125 antibody on the optical texture of DSCG is shown in Figure 6. Compared with the optical texture of BSA as presented in Figure 4, the brightness of the POM image for the anti-CA125 antibody was less intense, presumably due to the higher molecular weight of the anti-CA125 antibody (150 kDa) with respect to BSA (66.5 kDa), and thus a smaller amount of the anti-CA125 antibody was present at the LC–glass interface at the same mass concentration as BSA to induce the reorientation of DSCG. The difference in the level of disturbance may also be related to the interaction between DSCG and the analyte in an aqueous environment. It was reported that DSCG may bind to BSA through electrostatic interaction and alter the conformation of the protein [40], but how DSCG interacts with the anti-CA125 antibody remains to be investigated.

**Figure 7:**
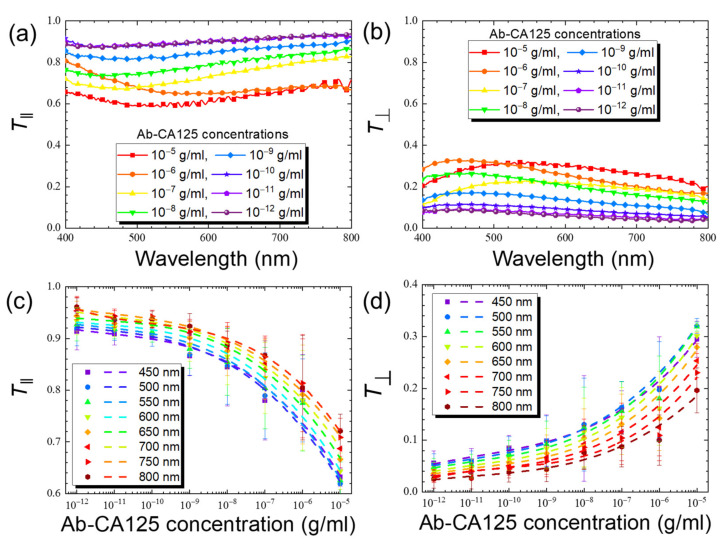
Reduced transmission data of LCLC with 14-wt% DSCG in the presence of the anti-CA125 antibody at various concentrations ranging from 10^−12^ to 10^−5^ g/mL. The reduced transmittance parameters (**a**) *T*_‖_ and (**b**) *T*_⊥_ in the wavelength range of 400–800 nm at various antibody concentrations and (**c**) *T*_‖_ and (**d**) *T*_⊥_ plotted against antibody concentration at selected wavelengths of 450, 500, 550, 600, 650, 700, 750, and 800 nm for quantitative purpose. *T*_‖_ (*T*_⊥_) corresponds to the measurements with parallel polarizers (crossed polarizers) for the spectrometric detection. Error bars represent standard deviations (*n* ≥ 3).

The transmission spectra obtained with parallel or crossed polarizers at various concentrations of the anti-CA125 antibody, as well as the results of the third-order polynomial regression of the calibration curve for the antibody, with *R*^2^ values ranging from 0.94 to 0.99, are illustrated in Figure 7. As seen in Figure 5 for BSA, the trends of the concentration-dependent decrease in *T*_‖_ and increase in *T*_⊥_ were also detected for the anti-CA125 antibody (Figure 7). However, the change in either reduced transmittance parameter with the concentration of anti-CA125 antibody was less pronounced in comparison with that for BSA, suggesting that the level of disturbance and the increase in *ϕ* caused by the antibody were less significant, in agreement with our observation in the optical texture (Figure 6). The LOD_‖_ calculated from Equation (6) for the anti-CA125 antibody based on the calibration curve in Figure 7c was higher than the LOD_⊥_ derived from Figure 7d at all selected wavelengths, with LOD_‖_ = 1.2 × 10^−10^ g/mL and LOD_⊥_ = 6.2 × 10^−12^ g/mL as determined at 450 nm.

**Figure 8:**
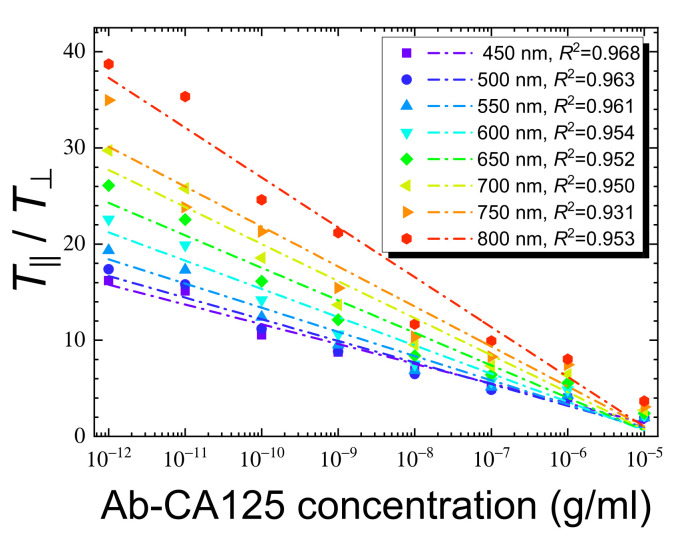
The correlation between the concentration of the anti-CA125 antibody and the ratio of reduced transmittance derived from Figure 7. The *T*_‖_/*T*_⊥_ ratios were calculated and plotted against the concentration of the anti-CA125 antibody at selected wavelengths of 450, 500, 550, 600, 650, 700, 750, and 800 nm. The coefficient of determination, *R*^2^, for each regression line is given in the legend as a measure of the agreement with the linear fit.

To further explore the anti-CA125 antibody data, the *T*_‖_/*T*_⊥_ values at various wavelengths were calculated and plotted against the concentration of the anti-CA125 antibody and a linear correlation resulted (Figure 8). The absolute value of *T*_‖_/*T*_⊥_ was significantly larger than *T*_‖_ or *T*_⊥_ alone, decreasing with increasing analyte concentration, and was higher at longer wavelengths. Transforming the nonlinear behavior in Figure 7c to a linear correlation in Figure 8 thus amplifies the optical signal and its variation with analyte concentration through data processing, especially at longer wavelengths, where the detection sensitivity was inferior when only *T*_‖_ was considered. This allows quantitative analysis to be performed at less optimized conditions such as longer wavelengths or in the presence of a small amount of analytes. The LOD calculated from Equation (6) for the anti-CA125 antibody based on the calibration curve at 800 nm in Figure 8 was 2.6 × 10^−11^ g/mL.

### 3.4. LCLC-Based Quantitative Immunoassay of the Cancer Biomarker CA125

In the LCLC-based CA125 immunoassay, the biomarker CA125 was captured by the anti-CA125 antibody immobilized at the LC–glass interface through the highly specific antigen–antibody immunoreaction. As demonstrated in the design of our previously reported LC-based CA125 immunoassays, the amount of the immobilized anti-CA125 antibody was optimized so that enough antibodies were present to interact with a wide concentration range of CA125 in the analyte without creating background signals or false-positive results [28,29,30,31]. In the LCLC-based immunoassay platform, a maximal concentration was tested and predetermined for the immobilization of the anti-CA125 antibody without interfering with the planar alignment of the LCLC. In accordance with the LOD values at 450 nm for the anti-CA125 antibody, two antibody immobilization concentrations, 10^−10^ and 10^−9^ g/mL, were chosen for comparative studies. As the volume of the antibody solution for immobilization was 30 μL, the total amount of the anti-CA125 antibody reacted with the glass substrate when a 10^−10^ or 10^−9^ g/mL solution was applied corresponds to 3 or 30 pg, respectively. The anti-CA125 antibody was immobilized on a circular area on the PI-coated glass substrate, whose entire surface was then reacted with 10^−12^–10^−5^ g/mL CA125 so that nonspecific binding to the rubbed PI surface can be easily detected outside the area containing the antibody (Figure 2h–k).

**Figure 9:**
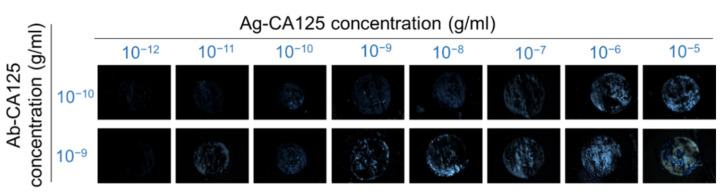
Optical textures of planarly aligned LCLC when the cancer biomarker CA125 was reacted with immobilized anti-CA125 antibody. The LCLC-based immunoassay was performed with an aqueous solution of 14-wt% DSCG as the sensing medium in contact with 10^−10^ or 10^−9^ g/mL anti-CA125 antibody immobilized on the PI-coated glass surface, followed by reaction with 10^−12^–10^−5^ g/mL CA125.

As shown in Figure 9, light leakage caused by the disturbance in the planar alignment of DSCG increased with increasing concentrations of CA125, and it became more significant at low CA125 concentrations when a larger amount of the anti-CA125 antibody was immobilized. The brightness of the optical texture was discernible at 10^−10^ g/mL (10^−11^ g/mL) of CA125 when 10^−10^ g/mL (10^−9^ g/mL) of the anti-CA125 antibody was immobilized, inferring that more CA125 immunocomplexes were formed when 10^−9^ g/mL anti-CA125 antibody was immobilized. When subjected to transmission spectrometric analysis at various CA125 concentrations, the general trend of *T*_‖_ decreasing and *T*_⊥_ increasing with analyte concentration remains (figure not shown), similar to that seen in Figure 5 and Figure 7 for BSA and the anti-CA125 antibody. In general, the LOD obtained at an anti-CA125 antibody concentration of 10^−9^ g/mL (LOD_9_) was smaller than that at 10^−10^ g/mL (LOD_10_), with LOD_9_ values obtained from the curves of *T*_‖_ and *T*_⊥_ at 450 nm versus CA125 concentration of 5.0 × 10^−11^ and 1.1 × 10^−10^ g/mL, respectively. Actually, at an optimized immobilization concentration of 10^−9^ g/mL anti-CA125 antibody, both the sensitivity and LOD of the LCLC-based CA125 immunoassay were improved in comparison with those obtained at 10^−10^ g/mL anti-CA125 antibody, without compromising the signal-to-noise ratio.

**Figure 10:**
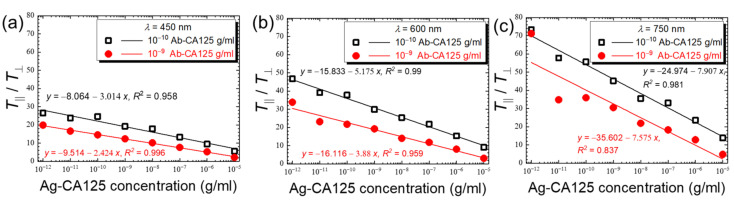
The correlation between CA125 concentration and the ratio of reduced transmittance at two immobilization concentrations of the anti-CA125 antibody, 10^−10^ and 10^−9^ g/mL. The ratios, *T*_‖_/*T*_⊥_, were calculated and plotted against the concentration of CA125 at selected wavelengths of (**a**) 450, (**b**) 600, and (**c**) 750 nm. The equation and coefficient of determination, *R*^2^, for each linear regression line are shown as a measure of agreement with the linear fit.

The ratios of reduced transmittance, *T*_‖_/*T*_⊥_, were calculated for both antibody immobilization concentrations and were plotted against CA125 concentration at representative wavelengths of 450, 600, and 750 nm, as shown in Figure 10. Similar to Figure 8 for the plots of *T*_‖_/*T*_⊥_ versus the concentration of the anti-CA125 antibody, a negative and linear correlation was found between the *T*_‖_/*T*_⊥_ ratio and the CA125 concentration, with the absolute values of *T*_‖_/*T*_⊥_ and the slope of the linear regression line increased with increasing wavelengths of the incident light (Figure 10). Because the *T*_⊥_ values obtained at 10^−10^ g/mL anti-CA125 antibody were generally lower than those at 10^−9^ g/mL anti-CA125 antibody, the absolute values of *T*_‖_/*T*_⊥_ at each CA125 concentration and the slope of the regression line became larger at a lower antibody immobilization concentration, when each pair of the CA125 calibration curves in Figure 10a–c was compared. These observations imply that, by converting *T*_‖_ to *T*_‖_/*T*_⊥_ through data processing, the initially less significant concentration-dependent response in Figure 7c at longer wavelengths or lower antibody immobilization concentrations can be enhanced, as seen in Figure 8 and Figure 10. The calculated LOD_10_ from the linear correlation between *T*_‖_/*T*_⊥_ and CA125 concentration at the more favorable 800 nm, a condition at which the LOD at an antibody concentration of 10^−10^ g/mL can be more accurately estimated, was 1.7 × 10^−10^ g/mL CA125.

In our previous biosensing studies based on thermotropic LCs, the lowest detectable BSA and CA125 concentrations were 10^−11^ g/mL BSA and 10^−8^ g/mL CA125 (with anti-CA125 antibody immobilized at 10^−7^ g/mL), respectively, when a nematic LC of high birefringence was employed as the sensing medium [28]. Through detection with a dye-doped LC in conjunction with transmission spectrometry, analyte concentrations as low as 10^−6^ g/mL BSA and 10^−5^ g/mL CA125 (with anti-CA125 antibody immobilized at 10^−7^ g/mL) can be discerned from the background [31]. In a single-substrate detection platform based on an LC-photopolymer composite film, signal amplification through photopolymerization gave rise to LOD values of 1.6 × 10^−12^ g/mL BSA and 2.1 × 10^−8^ g/mL CA125 (with anti-CA125 antibody immobilized at 10^−10^ g/mL), respectively [30]. In this study, the LOD_‖_ and LOD_⊥_ for BSA at 450 nm were 2.4 × 10^−10^ and 6.0 × 10^−11^ g/mL, respectively, whereas those for CA125 were 5.0 × 10^−11^ and 1.1 × 10^−10^ g/mL (with anti-CA125 antibody immobilized at 10^−9^ g/mL), respectively. The comparable and even lower LOD achieved by nematic DSCG demonstrates that LCLCs can serve as an alternative to thermotropic LCs as the biosensing media. Moreover, by exploiting the biocompatibility and hydrophilicity of DSCG, the end-point assay demonstrated in this study can be further transformed into real-time detection by mixing the target of detection (e.g., CA125) in the aqueous solution of DSCG and, after injection into the LC cell, monitoring the change in transmittance at a specific wavelength over time as the target protein associates with the immobilized capture molecule (e.g., anti-CA125 antibody).

## 4. Conclusions

In this study, a quantitative label-free biosensor for BSA protein assay and CA125 immunoassay was developed based on the spectrometric analysis of LCLCs. By employing the nematic phase of aqueous DSCG as the sensing mesogen, the biosensing platform was designed so that when the planar alignment of LCLCs was disrupted by biomolecules present at the LC-glass interface, a concentration- and wavelength-dependent optical signal was produced, which was analyzed by transmission spectrometry in the visible spectrum with parallel or crossed polarizers. The reduced transmittance obtained with parallel or crossed polarizers, *T*_‖_ or *T*_⊥_, was negatively or positively correlated to BSA concentration, respectively, thus enabling calibration curves to be constructed for protein quantitation. The LCLC-based CA125 immunoassay was established with an optimized antibody immobilization concentration of 10^−9^ g/mL anti-CA125 antibody, at which the sensitivity and the LOD were improved compared with those obtained at 10^−10^ g/mL anti-CA125 antibody. In addition, the linear correlation between the *T*_‖_/*T*_⊥_ ratio and the logarithm of CA125 concentration may offer more accurate quantitative results at longer wavelengths, where the detection sensitivity was lower when only *T*_‖_ was considered. The results from this study reveal a new perspective on how the nematic phase of LCLCs can be employed in LC-based biosensing similar to thermotropic LCs. By incorporating the water-soluble biomolecular analytes in the hydrophilic LCLCs, it is possible to further endow LCLC-based biosensors with real-time detection capabilities unattainable by hydrophobic thermotropic LCs.

## Data Availability

The authors confirm that the data supporting the findings of this study are available within the article.
